# Neuroepigenetic mechanisms in disease

**DOI:** 10.1186/s13072-017-0150-4

**Published:** 2017-10-16

**Authors:** Michael A. Christopher, Stephanie M. Kyle, David J. Katz

**Affiliations:** 1 Department of Cell Biology, Emory University School of Medicine, 615 Michael Street, Atlanta, GA 30322 USA; 2 Department of Molecular, Cell and Developmental Biology, University of California, Los Angeles, Los Angeles, CA 90095-7239 USA

**Keywords:** Neuroepigenetics, DNA methylation, Histone modification, Rett syndrome, Fragile X syndrome, Alzheimer’s disease, MECP2, FMR1, LSD1/KDM1A

## Abstract

Epigenetics allows for the inheritance of information in cellular lineages during differentiation, independent of changes to the underlying genetic sequence. This raises the question of whether epigenetic mechanisms also function in post-mitotic neurons. During the long life of the neuron, fluctuations in gene expression allow the cell to pass through stages of differentiation, modulate synaptic activity in response to environmental cues, and fortify the cell through age-related neuroprotective pathways. Emerging evidence suggests that epigenetic mechanisms such as DNA methylation and histone modification permit these dynamic changes in gene expression throughout the life of a neuron. Accordingly, recent studies have revealed the vital importance of epigenetic players in the central nervous system and during neurodegeneration. Here, we provide a review of several of these recent findings, highlighting novel functions for epigenetics in the fields of Rett syndrome, Fragile X syndrome, and Alzheimer’s disease research. Together, these discoveries underscore the vital importance of epigenetics in human neurological disorders.

## Introduction to neuroepigenetics

Advances in molecular biology and genomics have allowed researchers to uncover roles for epigenetic regulation in a diversity of biological processes previously unexplained by classical genetics. These epigenetic mechanisms manifest as a variety of chromatin modifications and states, altering gene function in a transient or persistent manner and acting as a vector for the passage of information. For example, considerable attention has been paid to the contributions of epigenetic aberrations during oncogenesis. In fact, epigenetic changes have been designated as one of the hallmarks of cancer [1–3]. Oncogenic phenotypes can arise when an insult or a mutation in an epigenetic pathway creates heritable epigenetic information that is passaged through mitosis. However, mature neurons no longer undergo mitosis and thus do not have an obligation to pass information onto daughter cells. This makes the developed nervous system an interesting system to study epigenetic regulation. What epigenetic regulation is required in post-mitotic neurons, and how is it established and maintained? Are there consequences for misregulation of these processes that manifest in human disease? If so, it will be important to determine the contribution of epigenetic mechanisms to complex diseases such as autism spectrum disorders (ASD) and neurodegenerative disorders, where identification of a purely genetic etiology has been elusive. In the first part of this review, we introduce our current understanding of epigenetic modifications, with an emphasis on what is known about them in post-mitotic neurons. In the second part of this review, we highlight how disruption of these processes may contribute to three specific examples of neurological disease. Importantly, this review is not intended as a comprehensive summary of all that is known about neuroepigenetics (for more information on neuroepigenetics, please see [4–6]. Rather, we hope that it will serve as an entry point for researchers from multiple fields to begin to consider how post-mitotic neurons may have unique epigenetic requirements, and how epigenetic mechanisms may contribute to neurological disease.

## Epigenetic modifications and their regulators

### DNA methylation

One level of chromatin modification is the methylation of DNA at cytosine residues and, as recently discovered, on adenosine residues. Given the size and complexity of the vertebrate genome, methylated DNA adds a layer of regulation that further refines the cellular transcriptional profile. Cytosine methylation in mammals occurs primarily in a CpG dinucleotide context in most cell types. It has been most extensively studied in the context of CpG islands: genomic regions containing a higher rate of CpG dinucleotides than the rest of the genome [7, 8]. These regions are thought to be evolutionarily conserved regulatory elements, as CpGs are underrepresented in the genome due to spontaneous deamination of methylcytosine into thymine, resulting in a T/G mismatch. If this mismatch is not detected as a mutation by the DNA repair pathway, it results in a C/G to T/A transition mutation in the resulting daughter strand [9]. Thus, preservation of CpGs at CpG islands is thought to occur via positive selection at evolutionarily conserved promoters. Cytosine methylation at CpG dinucleotides in promoters is correlated with repressed transcription, while cytosine methylation in gene bodies, as well as methylation of adenosines, appears to be correlated with an active transcriptional state in many cells [10–14]. A notable exception to this trend occurs within neurons, where cytosine methylation within gene bodies is anticorrelated with transcription [15, 16]. Cytosine methylation occurs by the addition of a methyl group to the fifth position of the cytosine base (5mC) and is donated by *S*-adenosylmethionine. This reaction is carried out by DNA methyltransferase (DNMT) enzymes. DNMT3a and DNMT3b can methylate cytosines de novo [17]. DNMT1 recognizes palindromic hemimethylated CpG dinucleotides and adds a methyl group to the unmethylated cytosine on the opposite strand [18]. Through the action of DNMT1, the maintenance methyltransferase, DNA methylation can be inherited through DNA replication and cell division, though it remains possible that DNMT1 has de novo methyltransferase activity in certain contexts.

However, this paradigm is challenged in the context of a post-mitotic neuron. Within the context of mature neurons, methylated CpG dinucleotides were once thought to be very stable; however, DNA methylation is dynamically regulated in the adult nervous system [19, 20]. This mechanism is crucial in synaptic plasticity and memory formation [21, 22]. Specifically, work from the Sweatt Lab found that non-specific inhibition of DNMT activity altered DNA methylation in the adult brain and changed the methylation landscape surrounding plasticity promoting genes such as brain-derived neurotrophic factor (BDNF) [23]. Remarkably, the group determined that DNA hypermethylation occurs in a locus-specific manner following a single associative learning experience. Further, pharmacological inhibition of DNA methylation abolished remote memory in rats [24]. In addition, Fan and colleagues have provided further evidence that mature neurons require active maintenance of DNA methylation. The researchers showed that when both *Dnmt1* and *Dnmt3a* are deleted in post-mitotic excitatory neurons, mice display learning and memory deficits without neuronal loss. This phenotype only occurred when both methyltransferases were deleted, suggesting that the enzymes redundantly regulate neuronal processes in adult excitatory neurons. Hippocampi from double *Dnmt1/3a* mutant animals displayed abnormal long-term potentiation following stimulation, suggesting the learning deficits are due to neuronal plasticity errors. Genes involved in immune response, cell communication, and mRNA transcriptional regulation were also aberrantly expressed in double mutant mice brains due to hypomethylation of their promoter regions. This study demonstrates that the DNA methyltransferases play redundant roles in post-mitotic neurons by regulating gene expression associated with neuronal homeostasis, which affects higher-order functions like learning and memory. Fan and colleague’s findings suggest that DNA demethylation occurs at promotor regions in the absence of *Dnmt1* and *Dnmt3a* [25]. This was surprising, as it suggests a role for *Dnmt1*, a methyltransferase that typically transmits DNA methylation to daughter cells, in the maintenance of DNA methylation in non-dividing adult neurons [25].

Recently, some DNA demethylation by-products have been described as biologically functional, suggesting that DNA methylation and demethylation are dynamic processes. 5mC can be oxidized to form 5-hydroxymethylcytosine (5hmC), 5-formylcytosine (5fC), and 5-carboxylcytosine (5caC) in sequential reactions carried out by the ten-eleven translocase (TET) family of enzymes [26, 27]. This series of oxidation reactions is thought to be a mechanism of active demethylation, rather than one of passive demethylation, which occurs through DNA replication [28]. The 5hmC mark is most highly enriched in the brain when compared to any other tissue [29–31], and TET1, which catalyzes the conversion of 5mC to 5hmC, is activated by neuronal activity [19]. Current evidence suggests that within the brain, 5hmC is acquired in a developmentally dependent manner, occurs exclusively in the CpG context, and is enriched in the gene bodies of highly expressed genes [16, 31, 32]. This suggests a role for 5hmC in active transcription within neurons [32]. Indeed, Sweatt and colleagues have shown that TET1 expression positively regulates subsets of genes associated with learning and memory [19]. Further, Rudenko et al. [33] found gross downregulation and hypermethylation of activity-related genes, including *Npas4, c*-*Fos,* and *Arc* in a TET1 null mouse model. Unsurprisingly, TET1 null animals display impaired synaptic plasticity and memory extinction. TET3 similarly regulates synaptic transmission in an activity-dependent manner. Knockdown or TET3 or inhibition of base excision repair, the process by which an oxidized cytosine is replaced with an unmethylated cytosine, elevates glutamatergic signaling in hippocampal neurons, whereas overexpression of TET3 decreases it [34].

5fC and 5caC are found at extremely low levels in the embryonic stem cell genome (at major satellite repeats), but accumulate in regulatory regions when thymine-DNA glycosylase (TDG) is depleted [35]. TDG removes the 5caC base to allow for base excision repair enzymes to replace the abasic site with an unmethylated cytosine [36]. This suggests there is dynamic demethylation of cytosines occurring in embryonic stem cells at regulatory regions. This could mean that 5fC and 5caC are simply reaction intermediates that can be captured, but are normally found at low levels at specific loci. Currently, their epigenetic role is unclear; however, a recent study showed that 5fC and 5caC reduce RNA polymerase II elongation rate and therefore may influence splicing [37].

Methylated cytosine in the context of CpG dinucleotides was historically thought to be the sole form of DNA methylation. However, it is now recognized that cytosine methylation does not occur exclusively in the CpG context [16, 38, 39]. Mapping of DNA methylation with single base pair resolution has found that it also occurs in a non-CpG context (termed 5mCH, where H is an A, T, or C) [40]. The highest levels of 5mCH have been observed in neurons and embryonic stem cells [14, 16]. This enrichment of 5mCH in these cell types suggests it may have cell type-specific roles. 5mCH is lost during embryonic stem cell differentiation and then reacquired by neurons during neuronal maturation [16, 39, 40]. 5mCH methylation requires DNMT3a for active maintenance in post-mitotic neurons [39], and 5mCH may play a critical role in the specification of neuronal subtypes. Recent work from Ecker, Nathans, and colleagues describes how the DNA methylome predicts current and previous cell type gene expression [41]. Using the INTACT method for nuclei purification [42], they isolated three different neuron subtypes (excitatory pyramidal neurons, parvalbumin interneurons, and vasoactive intestinal peptide interneurons) and performed RNA-seq along with MethylC-seq. This allowed for generation of matching transcriptome and DNA methylome datasets from the different neuronal subtypes. They found that gene body 5mCH correlates more strongly with repressed transcription across all three neuronal subtypes, more so than any other DNA methylation pattern (5mCH and 5mCG in promoters and 5mCG in gene bodies). This finding suggests that gene body 5mCH is tightly linked to transcriptional control beyond other contexts of DNA methylation in neurons and may be part of the epigenetic program that defines neuron subtype specification [41]. As 5CH methylation is acquired during neuronal differentiation, it raises the question: do aberrations in writing or reading this modification underlie neurological disease?

### Histone modifications

Histones are a highly conserved set of proteins that assemble in an octamer composed of pairs of H2A, H2B, H3, and H4. Following replication, DNA is first complexed with H3 and H4, and H2A and H2B are deposited to form nucleosomes [43, 44]. Histone proteins can be posttranslationally modified at their N-terminal tails, which are less structured than the core of the octamer. These histone modifications add another layer of epigenetic information and transcriptional control by affecting the three-dimensional structure of the chromatin [45, 46]. Importantly, these modifications are reversible, allowing for dynamic regulation of nucleosome organization and chromatin structure. For example, a subset of histone modifications is associated with tight nucleosome packaging which prevents transcription of genes at the locus, while other histone modifications relax the chromatin structure and allow the transcriptional machinery to access genes. The posttranslational modifications of histones are controlled by “writers” and “erasers.” Writers, like histone methyltransferases or acetyltransferases [47–49], establish histone modifications, whereas erasers, like histone demethylases and deacetylases, remove histone modifications [50, 51]. These chromatin modifications are then functionally recognized by “readers,” enzymes like chromatin remodelers that recognize certain modifications to manipulate chromatin [52–54]. This language of histone modifications and their influence on chromatin has been termed the “histone code” [55]. For a comprehensive review on histone posttranslational modifications, please see [56, 57].

A wide range of modifications have been described. Phosphorylation of histone tails appears to result from signal transduction pathways. For example, phosphorylation of serine 10 on histone 3 (H3S10p) is acquired in genes after response to stimulation, such as growth factors [58], and marks mitotic cells. Acetylation of lysine residues is thought to promote euchromatin formation by reducing the positive charge of lysine side chains, thereby disrupting the electrochemical attraction of the positively charged histones to the negatively charged backbone of the DNA. This can be functionally seen by the disruption of the 30 nm fiber (a proposed higher-order structure of chromatin) by H4K16 acetylation [46].

Methylation of specific histone–lysine residues also influences chromatin shape, but in a more complex manner. One of the most highly studied methylation marks is H3K4 methylation, which is associated with active transcription. H3K4 can be mono-, di-, or trimethylated by the mixed lineage leukemia (MLL) and SET family enzymes [59–62]. H3K4me3 is found primarily in the promoters of active and poised genes, while H3K4me2 is found in the gene bodies and enhancers associated with active genes. H3K4me1 is found in enhancers, as well as in promoters, and at the 3′ end of active genes [63]. Mono- and dimethylation of H3K4 is removed by the amine oxidase containing lysine-specific demethylase 1 (LSD1/KDM1A) and lysine-specific demethylase 2 (LSD2/KDM1B). However, amine oxidase demethylases are incapable of removing trimethylation. This is accomplished by a set of Jumonji domain (Jmj) containing demethylases, JARID1a/KDM5A and JARID1b/KDM5B [64, 65]. Another well-studied modification is H3K9me, which is associated with gene repression. Methylation of H3K9 is established by several methyltransferases, including SUV39H1, G9a (EHMT1), and SETDB1 (ESET) [66, 67]. H3K9 is primarily found di- and trimethylated and often coincident with DNA methylation in mammalian genomes. It has been proposed that H3K9 methylation and DNA methylation can be inherited together through cell division [68, 69]. H3K9 methylation is erased by the Jmj domain-containing JHDM2A/KDM3A [70]. Modification of H3K27 is primarily found in the form of trimethylation and is established by EZH2 of the polycomb repressive complex 2 (PRC2) [71]. It is erased by the Jmj-containing enzymes UTX/KDM6A and JMJD3/KDM6B [72, 73]. DNA methylation and H3K27me3 are mutually exclusive in the genome, but the significance of this relationship is unclear [74]. These examples highlight the complex interplay between histone methylation and other epigenetic regulators.

Studies have demonstrated that histone modifications and chromatin remodeling are required to facilitate dynamic, complex tasks such as synaptic plasticity, learning, and long-term memory formation. Genetic or pharmacological manipulation of histone acetylation and deacetylation results in a myriad of learning and memory impairments. Specifically, deletion of HDAC2 embryonically results in enhanced long-term potentiation (LTP) and fear conditioning, while overexpression impairs fear condition and spatial learning [75]. Deletion of HDAC2 postnatally in glutamatergic neurons similarly recapitulated these findings [76]. However, mice with a deletion of HDAC1 have no overt phenotypes suggesting that HDAC2 deacetylates in a memory and learning-specific capacity [75]. Alternatively, deletion of HDAC4 in brain results in impaired hippocampal-dependent learning and memory and long-term memory formation [77]. Further, haploinsufficiency of HDAC4 causes Brachydactyly mental retardation syndrome, a disorder characterized by severe learning and memory deficits [78]. These studies and findings suggest that different HDACs act as crucial positive and negative regulators of learning and memory. As such, use of non-specific pharmacological HDAC inhibitors should be approached cautiously.

### RNAs that regulate chromatin

In addition to histone modifications and DNA methylation, a further layer of epigenetic regulation exists at the level of RNA-mediated establishment and regulation of chromatin states. The first evidence of RNA-influenced chromatin modification in mouse was that of the X-inactive specific transcript (*Xist)* long-noncoding RNA [79, 80]. In placental mammals, female XX cells express the *Xist* transcript from the inactive X chromosome, leading to multiple copies of the transcript coating the chromosome in *cis* [81]. The *Xist* transcripts bind to and recruit the heterochromatin-forming PRC2 [82] and YY1 protein, silencing the inactive X chromosome [83]. Other long-noncoding RNAs have been shown to regulate chromatin in a similar manner. For example, the HOX transcript antisense RNA (HOTAIR) serves as a scaffolding molecule between the PRC2 complex and LSD1-CoREST complex [84]. By coupling these complexes, there can be a targeted simultaneous removal of active H3K4me by LSD1 and deposition of repressive H3K27me, serving as an epigenetic switch from on to off. Additionally, Piwi-interacting RNAs (piRNAs), short RNAs that interact with the PIWI family of proteins, are thought to specifically target transposon sites in the germline in order to facilitate silencing [85]. In all of these cases, a noncoding RNA serves as a trigger or guide for other molecules in the maintenance of the chromatin, adding another layer of complex epigenetic regulation.

**Fig. 1:**
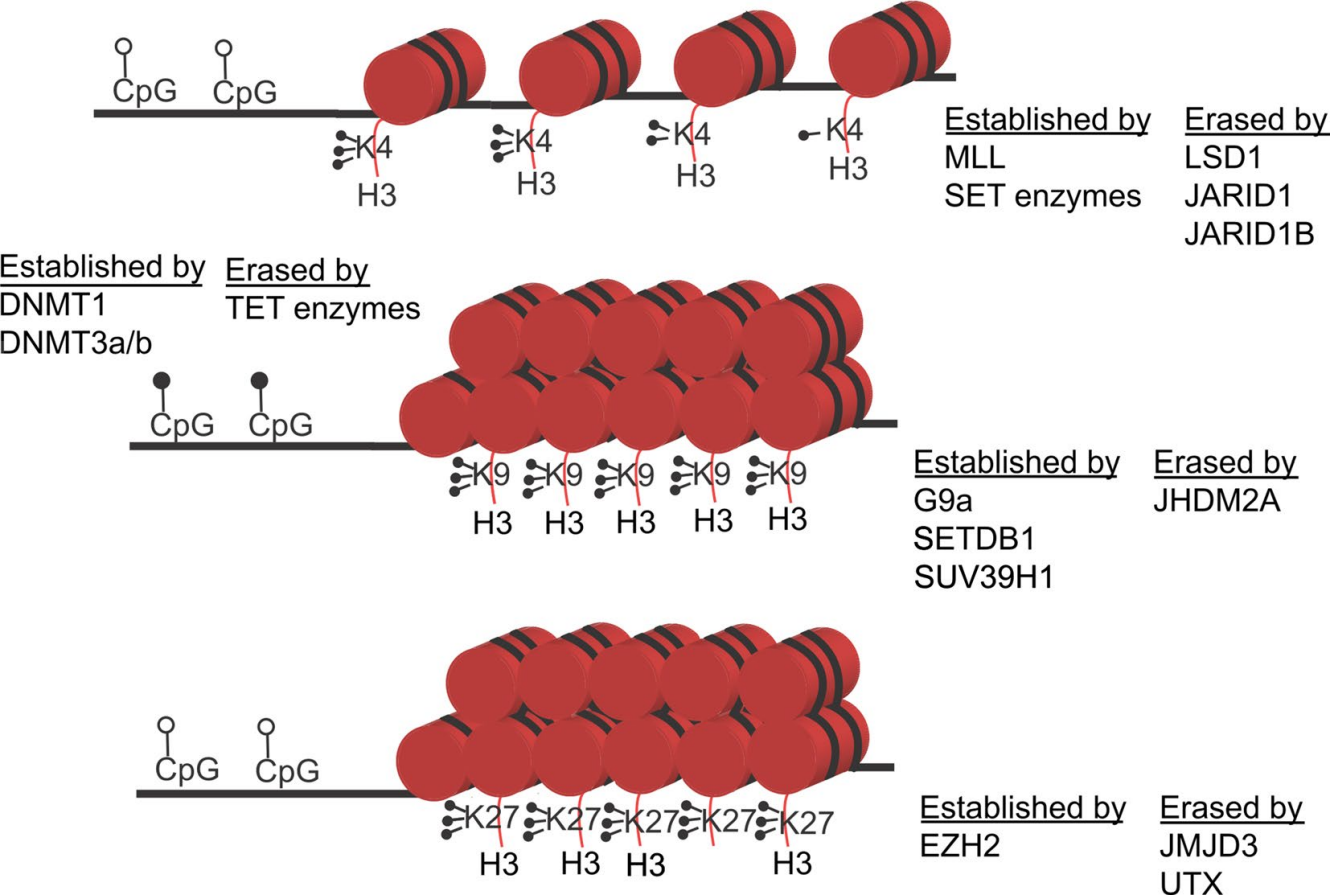
Chromatin modifications and their associated factors. Transcriptionally permissive chromatin is associated with the absence of DNA methylation (*open lollipops*) and the presence of H3K4me3/2/1. This mark is established by the MLL and SET family of enzymes and is found in genes actively undergoing transcription. H3K4me3 is found in the promoters, H3K4me2 is found in gene bodies, and H3K4me1 is found in the enhancers of active genes. These marks are erased by demethylases such as LSD1, JARID1, and JARID1b. Transcriptionally repressive chromatin features concurrent cytosine methylation (*closed lollipops*) and H3K9me3. Cytosine methylation is established by the DNA methyltransferases and erased by the TET family of enzymes. H3K9me3 is established by G9a, SUV39H1, and SETDB1 and erased by JHDM2A. Alternatively, transcriptionally repressive chromatin can contain H3K27me3 and no cytosine methylation (*open lollipops*). H3K27me3 is established by EZH2, part of the polycomb repressive complex, and is erased by JMJD3 and UTX

Together, many different facets of epigenetic regulation dynamically influence the state of chromatin, fine-tuning transcriptional programs that establish different cell fates (Fig. 1). During neuronal development and in mature neurons, it is increasingly clear that defects in these epigenetic mechanisms underlie neural disease. Below, we highlight three specific emerging examples of these mechanisms.

## MeCP2 regulates long gene expression in neurons

One protein that plays a role in epigenetic-mediated neurological disease is the X-linked methyl-CpG-binding protein 2 (MeCP2). It was first identified in an assay for proteins that bind methylated DNA, where it showed a much higher affinity for oligonucleotides containing methylated cytosine than the previously described MeCP1 [86]. While MeCP1 required at least 12 methylated cytosines per oligonucleotide for recognition, MeCP2 demonstrated binding capabilities with just a single, symmetrically methylated CpG site. Given the long-standing theory that site-specific DNA methylation is a transcriptionally repressive mark, it was immediately speculated that MeCP2 acts as a transcriptional repressor by binding methylated CpGs to inhibit transcription [86]. Indeed, in vitro, MeCP2 was found to repress transcription of reporter genes in a methyl-DNA-dependent manner through its association with Sin3a, a transcriptional repressor, and histone deacetylases (HDACs), which facilitate chromatin compaction [87–89]. Transcriptional repression associated with MeCP2 is relieved by treatment with the HDAC inhibitor trichostatin A, suggesting that MeCP2-mediated HDAC recruitment to methylated DNA is a critical step in MeCP2-mediated repression [90].

MeCP2 is ubiquitously expressed throughout all human and mouse tissues, but is most highly abundant in the brain. In neurons, its expression levels are comparable to histone octamers in adult animals [91]. Within the central nervous system, MeCP2 is expressed at low levels during neurogenesis, but gradually increases during neuronal maturation and synaptogenesis, reaching peak expression in mature, post-migratory neurons. This suggested a potential role for MeCP2 in the maintenance of neuronal maturation, activity, and plasticity [92–94]. Loss-of-function mutations in MeCP2 are the cause of Rett syndrome (RTT) [95], a devastating neurological disorder. As MeCP2 is an X-linked gene, RTT primarily affects females, since males with loss-of-function mutations in MeCP2 typically die in utero or perinatally [96]. Following a period of seemingly normal development during the first 6–18 months of life, RTT patients undergo a period of developmental stagnation followed by rapid, progressive motor deterioration and neurological regression. Patients also display a characteristic hand wringing and autism spectrum-like behavior. The onset of symptoms in RTT patients occurs at the same developmental timepoint that MeCP2 expression normally increases in the unafflicted brain. Despite decades of intense research on MeCP2, there is still no clear mechanism as to how MeCP2 dysfunction could lead to RTT; however, patient mutations tend to cluster in the methyl binding domain and transcriptional repressor domains of MeCP2, suggesting that transcriptional repression is inhibited in patients [97].

**Fig. 2:**
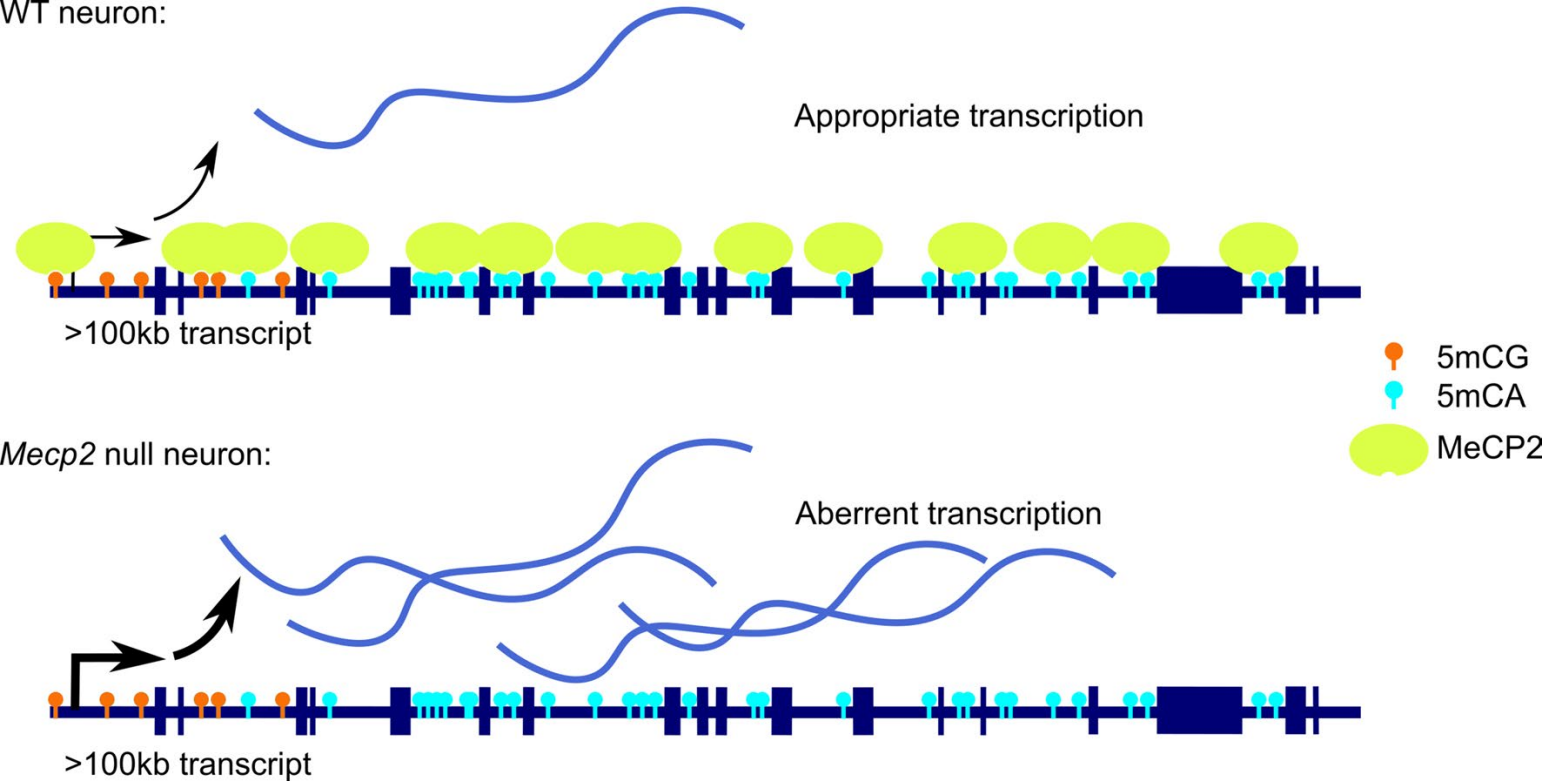
MeCP2 regulates long gene expression in a 5mCA-dependent manner. The 5mCG mark is commonly found enriched at gene promotor regions, while the 5mCA mark is enriched in the gene bodies of exceptionally long genes (>100 kb) in neurons. The epigenetic “reader” MeCP2 binds both marks, but has a strong affinity for 5mCA in long gene bodies. In wild-type neurons, this interaction represses transcription of long genes and may allow for fine tuning of gene expression. Currently, the machinery and mechanism by which MeCP2 silences long genes is unknown. In the *Mecp2* null neuron, 5mCA marks in long genes go unrecognized and the locus is aberrantly transcribed

Intriguingly, the developmentally regulated increase in MeCP2 expression within the CNS also coincides with the acquisition of 5mCH in neurons. Indeed, in addition to its interaction with methylated CpG residues, MeCP2 has been reported to regulate gene transcription through its capacity to bind methylated CA dinucleotides (5mCA) in vitro and in mouse brain [16, 39, 98, 99]. Recently, Gabel et al. [100] described a mechanism by which neurons regulate the expression of very long genes (e.g., >100 kb) in a MeCP2- and 5mCA-dependent mechanism. Specifically, they found that in brains of RTT patients and in mouse models of RTT, there is a genome-wide length-dependent increase in gene expression. The authors further show that MeCP2 binds 5mCA with high affinity and that MeCP2 binding is enriched in gene bodies with a high level of 5mCA, suggesting that MeCP2 binds 5mCA residues to repress transcription (Fig. 2). Notably, long genes that contain high levels of 5mCA are highly associated with neuronal processes, raising the possibility that their expression must be tightly regulated in neurons by MeCP2. Consistent with this model, disruption of *Dnmt3a* leads to a length-dependent increase in expression of these genes, presumably through loss of 5mCA. This role for MeCP2 as a regulator of long genes appears to be a neuron-specific phenomenon, as other tissues do not display this misregulation of long gene expression. This work suggests that MeCP2 tempers the expression of neuronal-specific long genes to prevent their overexpression, which could disrupt neuronal physiology [100].

Given the pattern of gene body 5mCH in neurons [16, 41] and the role for MeCP2-mediated repression of long genes [100], the Greenberg Lab has proposed a novel hypothesis to address the underlying cause of RTT pathology: as neurodevelopment takes place, neuronal subtypes are specified, and gene body 5mCA is acquired at genes where expression either needs to be repressed or tightly regulated. Normally, this gene body methylation is written by DNMT3a and read by MeCP2 and associated factors. However, in the absence of MeCP2, this finely tuned regulation is lost and derepression of neuronal genes occurs. In this model, perturbation of DNMT3a could lead to aberrant 5mCA, and thus altered MeCP2 binding, and may itself lead to neurological dysfunction. In both scenarios, loss of regulation mediated by DNA methylation can then lead to inappropriate transcriptional regulation of neuronal genes, causing neuronal dysfunction and severe autism-like phenotypes. Notably, disruption of DNMT3A has recently been reported as the underlying cause of the neurodevelopmental disorder Tatton–Brown–Rahman syndrome [101], and mutations in DNMT3A have been identified in individuals diagnosed with autism spectrum disorders [102]. Thus, disruption of gene regulation by 5mCA may be a common site of disruption across neurodevelopmental disorders.

## FMR1 mRNA induces epigenetic silencing of the locus

A role for RNA-mediated epigenetic regulation and DNA methylation-dependent silencing in neurological disease can be found in Fragile X syndrome (FXS), the most common form of inherited intellectual disability. FXS patients have characteristic IQ scores below 70 and are commonly on the autism spectrum [103]. The disease is named for the appearance of bent X chromosomes on karyotypes of patients. This bend takes place at the Fragile X mental retardation 1 locus (*FMR1*), and the subsequent silencing of this locus causes FXS [104]. The disease is most often observed in males because they are hemizygous for the gene and are therefore susceptible to mutations in their single allele. The protein encoded by the *FMR1* gene is Fragile X mental retardation protein (FMRP), an RNA binding protein that is proposed to tightly regulate local translation in neurons by inhibiting translation presynaptically [105, 106]. The etiology of the disease is thought to stem from an epigenetic silencing of the locus and consequential loss of the FMRP protein, increasing protein translation at synapses [107–110]. The *FMR1* locus features a CGG trinucleotide repeat in the 5′UTR, adjacent to the promoter. Individuals with 5–40 repeats are considered normal, while those with greater than 200 repeats develop FXS [111]. Those with 40–200 repeats are classified as having a premutation for the disease. Interestingly, a subset of these individuals develops a late-life gait ataxia and intention tremor known as Fragile X-associated tremor/ataxia syndrome (FXTAS) [112]. In contrast with those with the full mutation, FXTAS patients appear to have an overabundance of *Fmr1* transcripts [113]. It is unclear why premutation repeats escape silencing in favor of increased expression and how exactly this leads to the condition, but there is a clear toxicity due to overexpression of the *Fmr1* gene. This contrast with FXS, where the transcript is silenced, demonstrates the sensitivity of neurons to the dosage of the *Fmr1* gene and the complex epigenetic mechanisms that regulate the locus.

**Fig. 3:**
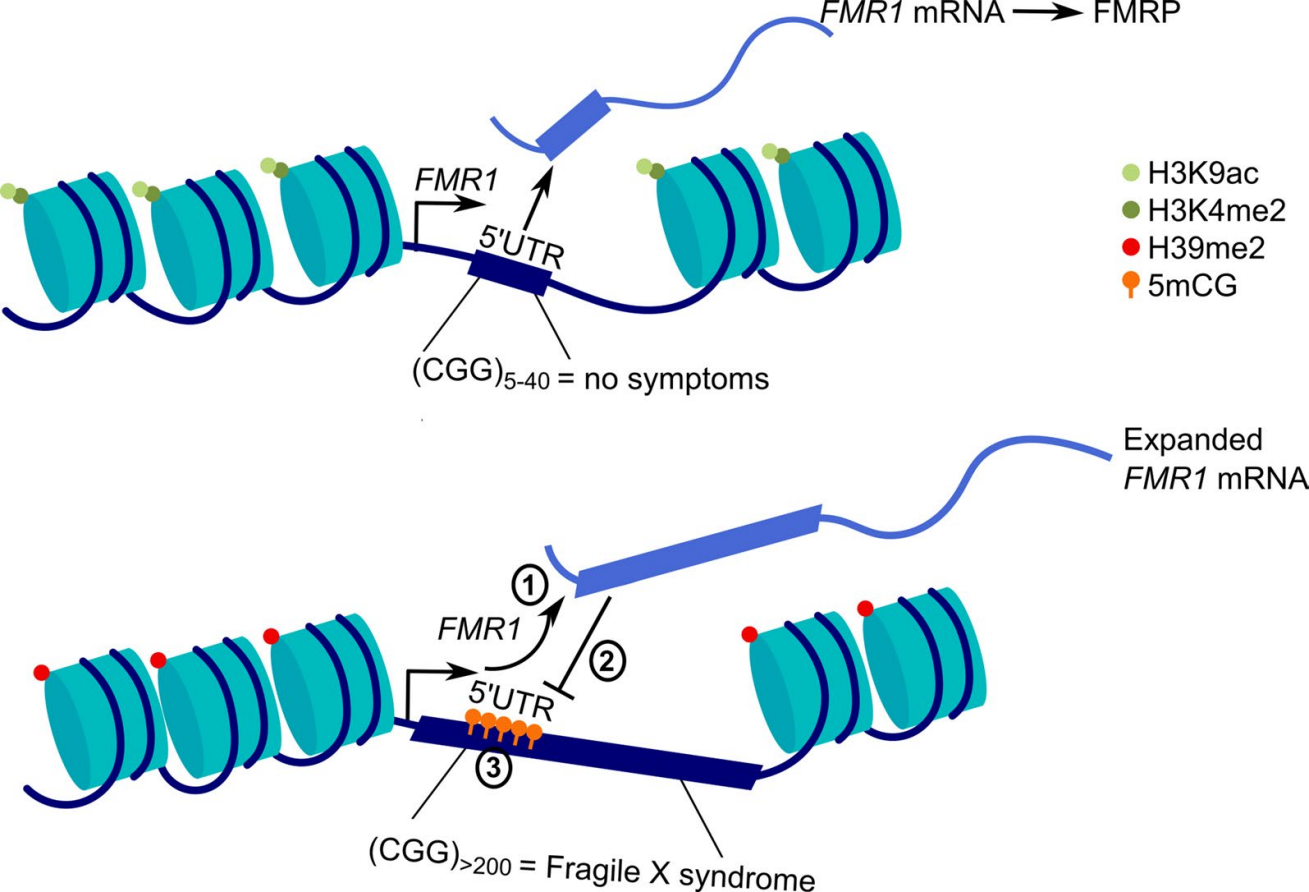
Expanded *FMR1* mRNA silences the *FMR1* locus through an epigenetic mechanism. The *FMR1* locus features a CGG trinucleotide repeat in the 5′UTR of the gene. Typically, the trinucleotide is repeated 5–40 times; however, expansion of this site to >200 repeats causes Fragile X syndrome. The trinucleotide repeat in the resulting expanded *FMR1* mRNA transcript (1) binds to its CGG expansion at the DNA locus to form a heteroduplex. Through an unknown mechanism, this interaction silences transcription from the *FMR1* locus (2). The locus then acquires a repressive chromatin state. Specifically, active histone marks H3K9ac and H3K4me2 are removed, while repressive mCG and H3K9me2 marks are added (3)

It was hypothesized that the CpG dinucleotide within the CGG repeat underlies the cause of FXS as CpG methylation could play a role in the silencing of the locus. Human embryonic stem cells with >200 CGG repeats express *Fmr1* in their undifferentiated state and lack chromatin modifications associated with silencing at the genetic locus. However, similar to what is observed in FXS embryos, when the cells are cultured under differentiation conditions, there is a downregulation of the transcript, acquisition of CpG DNA methylation, loss of H3K9 acetylation, and gain in H3K9 methylation at the *Fmr1* locus (Fig. 3) [114].

The factors that trigger silencing of the *Fmr1* locus were unknown until recent work from Jaffary and colleagues suggested that the *Fmr1* mRNA may initiate silencing of the locus [115]. Human embryonic stem cells (hESC) derived from FXS patients with a CGG expansion of at least 200 repeats display *Fmr1* expression comparable to undifferentiated control cells, but the transcript is silenced when the hESC are differentiated into neurons. During silencing, H3K4me2 is lost and H3K9me2 is gained at the *Fmr1* locus. Knockdown of the FMR1 transcript during differentiation prevented the acquisition of heterochromatin marks at the locus, suggesting the transcript itself may be necessary for the induction of heterochromatic silencing. The authors hypothesized that the CGG repeat in the transcript binds to the DNA to form a heteroduplex. Using chromatin isolation by RNA purification (ChIRP), which uses biotinylated oligonucleotides to immunoprecipitate RNA bound to chromatin, the authors showed that the *Fmr1* transcript binds to the *Fmr1* locus prior to heterochromatic silencing specifically in FXS cell lines. This interaction is most enriched when oligonucleotides are tiled next to the trinucleotide repeat of the transcript and when primer pairs closest to the trinucleotide repeat on the chromosome are used to amplify the precipitated DNA. This suggests the anchor point of the interaction takes place between the repeat regions of the DNA and RNA. Furthermore, the interaction is seemingly independent of protein intermediates and is abolished when treated with RNaseH, which selectively degrades RNA–DNA heteroduplexes. These data suggest that the trinucleotide repeat in the 5′-UTR of the transcript binds to the trinucleotide repeat of the DNA prior to the heterochromatic silencing of the *Fmr1* gene and leads to its silencing [115]. Future studies will likely focus on uncovering the machinery that responds to this RNA–DNA interaction to silence the locus and whether abolishing this interaction could be a therapeutic target.

## REST in neurodegenerative diseases

Alzheimer’s disease (AD) is the most common form of dementia worldwide, accounting for about 60–70% of cases [116]. Patients present with memory loss, disorientation, language defects, and behavioral changes and require years of care following cognitive decline. AD is characterized by a global reduction in brain mass during the course of the disease, primarily driven by loss of synaptic connectivity and neuronal processes. In addition, there is neuronal loss that begins in the excitatory neurons of the entorhinal cortex and moves to the dentate gyrus, CA3, and CA1 regions of the hippocampus, ultimately becoming widespread in many cases. Loss of synaptic connectivity has a greater correlation with cognitive decline than neuronal loss, suggesting it may play a greater role in the progression of AD [117]. In addition, improper neural network activity, hippocampal hyperactivity, and reduced hippocampal volume have been observed [118, 119]. Together, the combination of neuronal loss, neuronal hyperactivity and synaptic loss leads to cognitive dysfunction, resulting in the dementia phenotype.

During the course of the disease, there is an accumulation of pathological protein aggregates in the brain. Two distinct structures occur: senile plaques containing amyloid beta (Aβ) and hyperphosphorylated Tau containing neurofibrillary tangles [120, 121]. Aβ plaques are composed of a peptide derived from amyloid precursor protein (APP), a constitutively expressed transmembrane protein found in many cell types [122]. As part of normal neuron physiology, APP is cleaved by a series of secretases, α-secretase, β-secretase (BACE), and γ-secretase (Presenilin 1 and 2) [123–125]. Cleavage by BACE, then by the Presenilins, results in the 42 amino acid peptide product (Aβ42) [126]. Aβ42 occurs normally but also has a propensity to aggregate. Over decades, this leads to the formation of amyloid plaques. Unsurprisingly, mutations in this processing pathway are associated with familial forms of AD [127, 128]. In these cases, affected individuals develop early-onset AD, presumably due to a shift in APP cleavage that produces more Aβ42. However, in contrast, some healthy elderly individuals have buildups of protein aggregates, but do not show signs of cognitive impairment, suggesting that these aggregates are not entirely sufficient to cause neurodegeneration [129]. These observations exemplify the uncertainty surrounding the contribution of Aβ plaques to AD pathology.

Pathological aggregates of the microtubule-associated protein Tau are also highly correlated with AD [130]. Tau is normally found in the dendrites of neurons and facilitates the polymerization of microtubules. However, Tau protein can become hyperphosphorylated and acetylated resulting in aggregation of the protein into filamentous neurofibrillary tangles (NFTs) [131–133]. Formation of NFTs is thought to occur secondarily to accumulation of Aβ, as mutations in APP and Presenilins are sufficient to induce formation of both Aβ plaques and NFTs [134]. However, Tau aggregation in demented brains has a higher correlation with cognitive decline than the presence of Aβ plaques, suggesting Tau burden could be more tightly linked to the pathogenicity of the disease [130]. NFTs accumulate intracellularly and were originally thought to contribute to the disease by reducing the available pool of Tau to perform its normal function with microtubules, thereby disrupting cytoskeletal physiology [135]. This view has recently been challenged by two findings. First, loss-of-function mutations in Tau cause no gross abnormalities in mice [136]. Second, pathogenic hyperphosphorylated Tau accumulates in dendrites and can disrupt synaptic transmission [137–140]. These results raise the possibility that pathological aggregates of Tau could disrupt neurons by interfering with the function of other proteins.

Despite our understanding of which molecules aggregate and how it occurs, there is still a gap in understanding of how these aggregates cause neuronal cell death. Numerous models for the connection between protein aggregates and neuronal cell death have been suggested, including neuroinflammation mediated by microglia and the complement cascade [141, 142], cell cycle reactivation and DNA replication [143], nuclear pore instability [144], and loss of mitochondria coupled to the generation of reactive oxygen species [145]. Additionally, there have been several attempts to identify other agents using genetic approaches. One such gene with associations to AD is *ApoE.* ApoE is an apolipoprotein with three major allelic isoforms: ε2, ε3, and ε4. The isoforms arise from single amino acid substitutions. Individuals homozygous for the ApoE4 allele have a greater than 90% chance of developing late-onset AD [146]. This high incidence of AD in individuals with the mutation strongly suggests the ApoE protein has a role in the development of the disease; however, no clear role has yet been defined. Despite these clear genetic links with AD, identified genetic associations account for only a small fraction of susceptibility to AD, suggesting that other factors also contribute to the development of AD [147].

The RE1-silencing transcription factor (REST) was recently shown to be neuroprotective and aberrantly associated with protein aggregates during the course of neurodegenerative diseases. Thus, REST could be part of the missing piece in the AD puzzle [148]. REST, also known as neural restrictive silencing factor (NRSF), is a repressive transcription factor that binds to the canonical RE-1 recognition motif sequence and with the help of a set of corepressors silences transcription of neuronal genes in non-neuronal lineages [149, 150]. This silencing is achieved through the recruitment of HDACs and repressive histone methyltransferases, such as G9a the H3K9 methyltransferase [151]. Other corepressors that have been reported to associate with REST include CoREST, LSD1, MeCP2, and the C-terminal binding protein (CtBP) [152, 153]. Despite its status as a master negative regulator of the neuronal cell fate, REST is expressed at low levels in differentiated neurons, suggesting that it may still play a role in adult neurons.

The role of REST in adults is largely unexplored. However, Yankner and colleagues recently showed that REST expression is reactivated in healthy aging human neurons and gains neuroprotective properties by binding to and repressing apoptotic genes [148]. In mice, the authors showed that REST deficiency causes slight age-related neurodegeneration. In addition, mutants in *spr*-*4,* the *C. elegans* orthologue of the mammalian REST, were found to be more susceptible to oxidative stress and Aβ toxicity, further demonstrating the conserved neuroprotective qualities of REST. Yankner and colleagues also provided evidence that REST protein may be sequestered away from neuronal nuclei into autophagosomes along with protein aggregates that develop during the course of the disease in human brains derived from AD, frontotemporal dementia (FTD), and Lewy body dementia (LBD) cases. When REST protein is absent from the nucleus, there is an increase in global H3K9 acetylation levels, suggesting that epigenetic derepression occurs in the absence of REST. The degree of sequestration outside of the nucleus correlates with the severity of the cognitive impairment of the patient. These findings suggest that pathological Aβ protein aggregates may cause the mislocalization of REST, leading to the loss of its neuroprotective properties and normal epigenetic regulation. In the absence of REST protein, pro-apoptotic REST targets could be inappropriately expressed and lead to neuronal cell death by activating apoptosis pathways. Presumably, in the absence of nuclear REST, other corepressors dependent upon REST for targeting to specific loci may also be mistargeted, resulting in further misexpression. This cascade of aberrant transcriptional regulation may explain the large number of loci that become dysregulated in REST mutants.

## LSD1 is an epigenetic eraser and regulator of cell fate transition

As discussed earlier in this review, histone methylation can be reversed by histone demethylases, allowing the histone mark to serve as a dynamic regulator of transcription. The histone demethylase LSD1 specifically demethylates mono- and dimethylation of lysine 4 on histone H3 (H3K4me1/2) marks [51, 154] and requires the corepressor CoREST to demethylate H3K4 in the context of a nucleosome [155]. By demethylating H3K4, LSD1 acts as a transcriptional repressor by removing an active modification. Alternatively, when associated with the androgen receptor complex, LSD1 has been shown to demethylate H3K9me2 [156], allowing it to also serve as a transcriptional activator by removing a repressive mark.

The most studied role for LSD1 is as a facilitator of cell fate transitions. The mutants in the *C. elegans* LSD1 orthologue *spr*-*5* exhibit transgenerational progressive sterility over the course of 30 generations. This sterility is thought to arise from the accumulation of H3K4me2 in spermatogenesis genes, causing inappropriate expression of sperm genes in oocytes [157]. This role has led to the proposal that H3K4me2 acts as an epigenetic memory of transcription, and its passage through the germline can influence gene expression in offspring. A similar sterility phenotype has also been observed in *Drosophila* mutants for *Lsd1*, although sterility occurs in the first generation [158–160]. The function of LSD1 in regulating H3K4me2 is conserved in mice. Deletion of LSD1 from the female oocyte alone results in embryos arresting at the one- or two-cell stage. Arrested embryos feature altered DNA methylation patterns and failure to undergo the maternal-to-zygotic transcriptional transition [161, 162]. Together, these observations suggest that LSD1 functions in the reprogramming of epigenetic information between generations, likely by demethylating histones in the early zygote and preventing the passage of epigenetic cell fate information from one generation to the next.

LSD1 is also necessary for proper differentiation of mouse embryonic stem cells (mESCs) [163]. LSD1 binds to promoters and enhancers of critical embryonic stem cell genes in (mESCs), but does not actively demethylate the H3K4 methylation associated with these loci until the mESCs undergo differentiation. At this point, LSD1 erases the H3K4 methylation associated with these loci in coordination with the nucleosome remodeling and deacetylase complex (NuRD complex), which contains HDAC1 and 2, as well as the ATPase remodeler Mi-2β. When mESCs are differentiated in the presence of an LSD1 inhibitor, there is an inappropriate retention of critical stem cell gene expression and associated H3K4 methylation at both the enhancers and promoters of these loci. This suggests that LSD1 is responsible for the demethylation of H3K4 methylation at stem cell genes during mESC differentiation, which enables proper repression of mESC-specific loci [163]. As such, LSD1 regulates the cell fate transition of mESCs. A similar model has been proposed in murine hematopoietic stem cells [164].

The requirement of LSD1 to facilitate cell fate transitions is highly critical to many developmental processes. Consistent with this, depletion of LSD1 causes severe phenotypes in mice. *Lsd1* homozygous null mice arrest at embryonic day 5.5 and fail to properly elongate the egg cylinder before being resorbed by embryonic day 7.5 [165, 166]. In addition, loss of LSD1 in specific cell types causes a wide range of phenotypes. LSD1 depletion in the developing telencephalon causes defects in olfactory receptor choices. Mutation of a critical LSD1 phosphorylation site alters murine circadian rhythm. In vitro, researchers observe that LSD1 knockdown causes defects in plasma cell and hematopoietic cell differentiation; in vivo, deletion of *Lsd1* in pituitary tissue, testis stem cells, and trophoblast stem cells leads to differentiation defects. In addition, transgenic mice overexpressing LSD1 exhibit increased oxidative phosphorylation and fat, as well as paternally inherited transgenerational effects [162, 164, 165, 167–175].

## LSD1 promotes neural differentiation

Multiple lines of evidence also suggest an important role for LSD1 in neural differentiation. The LSD1/CoREST complex acts as a potent mediator of pyramidal neuron development by orchestrating radial migration. Inhibition of the LSD1/CoREST complex in embryonic mice results in a dramatic reduction of neural migration, causing newborn neurons to linger in the ventricular zone and subventricular zone for a longer period of time while retaining a multipolar shape characteristic of intermediate progenitor cells. This LSD1/CoREST-mediated migratory pathway appears to occur independently of REST activity [176].

**Fig. 4:**
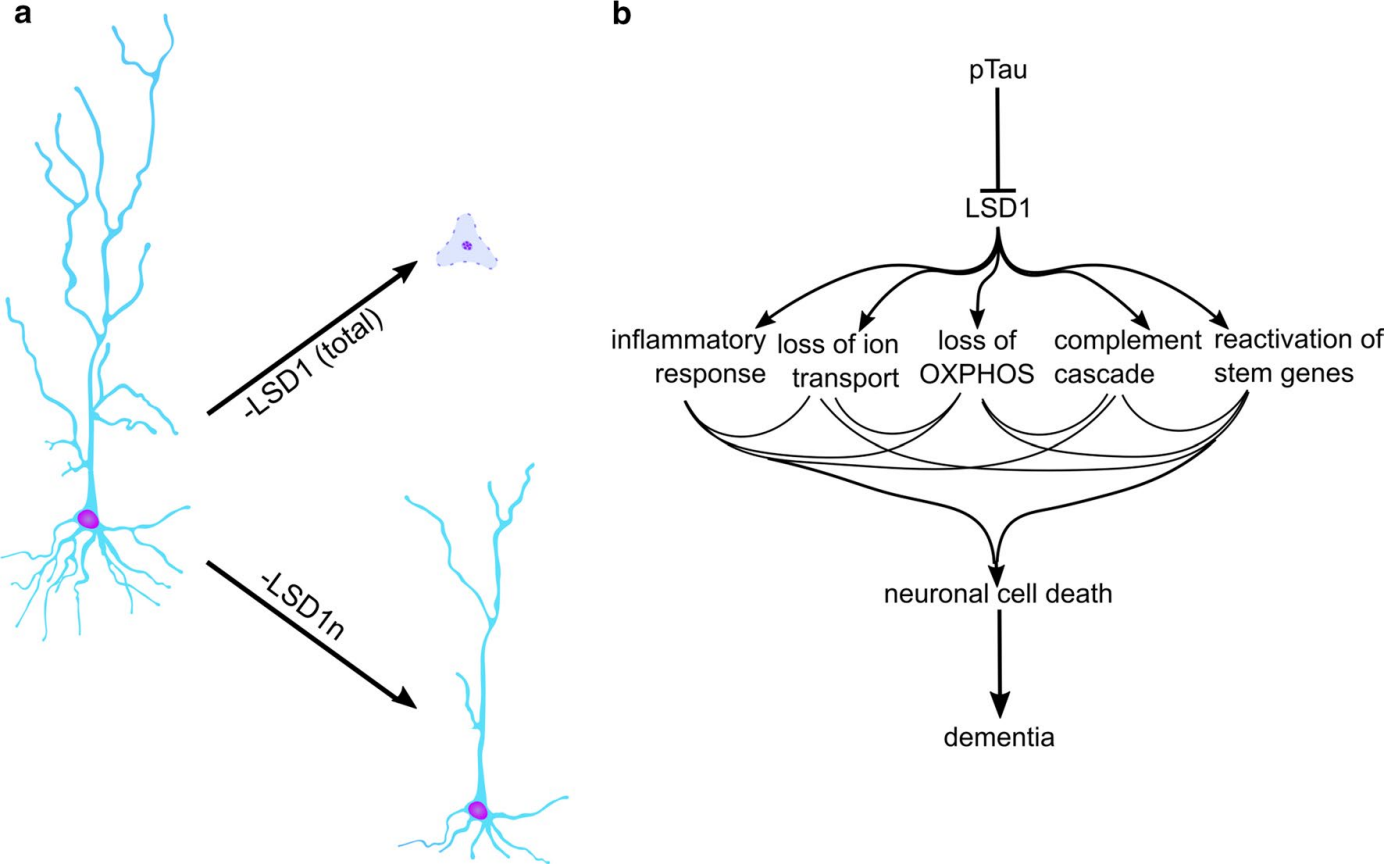
LSD1 is indispensable for neuronal health. **a** Loss of the neuronal-specific LSD1 isoform (LSD1n) reduces neurite length, branching, and width. LSD1n-specific null neurons are also hypoexcitable, and mice have a decreased susceptibility to seizures. Alternatively, loss of the entire LSD1 transcript in adult mice causes severe, rapid neurodegeneration as demonstrated by loss of neurites and pyknosis of affected nuclei. Cell death primarily occurs in the hippocampus and cortex. LSD1 mutant mice develop learning and memory deficits and die within 8 weeks post-deletion. **b** A model for LSD1 in Alzheimer’s disease emerges. As pathological hyperphosphorylated Tau tangles form in aging or sick neurons, LSD1 protein is sequestered in the cytoplasm, which inhibits its function as a histone demethylase. In the absence of nuclear LSD1, the complement cascade, microglial inflammatory response, and pluripotency-associated stem pathways become upregulated, while genes associated with ion transport and oxidative phosphorylation (OXPHOS) are downregulated. Though it is currently unclear which pathways are directly detrimental to cell health and which become perturbed as secondary effects, these aberrations cause neurons to die, leading to dementia in patients

Recently, a neuronal-specific isoform of LSD1 (LSD1n), which contains the additional exon 8a, has been implicated in regulation of neuronal differentiation. Specifically, this isoform demethylates the repressive H3K9me2 mark with its binding partner supervillin (SVIL) to activate expression of neuronal-specific genes during differentiation. Knockdown or mutation of the LSD1n isoform during induced in vitro differentiation decreases neurite outgrowth, while overexpression of intact LSD1n increased neurite outgrowth (Fig. 4a) [177, 178]. This neuronal isoform has also been shown to demethylate the repressive mark H4K20me2, activating transcription by promoting transcriptional initiation and elongation in response to neurotransmission [179]. In addition, LSD1n deficient mice display cognitive deficits and impaired spatial learning.

Maternal deposition of LSD1 in the developing oocyte adds yet another layer of complexity to the role of LSD1 in neural development. Partial loss of maternally loaded LSD1 causes in a hypomorphic phenotype in the resulting embryo. The few animals that survive the absence of maternal display developmental defects and behavioral abnormalities reminiscent of autistic-like phenotypes [162]. These findings for a role of LSD1 in neurodevelopment are bolstered by the recent discovery of three human patients with mutations in the *Lsd1* gene that display neurodevelopmental delay and intellectual disability [180].

## LSD1 is required for adult neuronal survival

Although LSD1 plays a clear role as a facilitator of cell fate transitions, little is known about the requirement for LSD1 in differentiated cell types. The importance of understanding this novel role has been emphasized by our recent discovery that LSD1 is required for neuronal survival and maintenance of adult, post-mitotic neuronal identity [181]. To assay its function in adult neurons, we deleted *Lsd1* using a tamoxifen-inducible CAGG-Cre (hereafter referred to as *Lsd1* ^*CAGG*^) in mice ranging from 2 to 6 months of age. Four weeks after *Lsd1* deletion, mutant mice have significantly reduced spatial learning, poor reference memory capacity, and impaired contextual fear conditioning. Approximately 7 weeks after tamoxifen injection, *Lsd1* ^*CAGG*^ mice undergo severe, rapid neuronal cell death, which primarily affects the hippocampus and cerebral cortex (Fig. 4a). At this point, mutant mice have severe motor deficits including lethargy, limb weakness and clasping, and the inability to maintain posture. Despite these severe motor phenotypes, the peripheral nervous system does not appear to be affected by CAGG-mediated *Lsd1* deletion. By 8 weeks post-injection, mutant mice are severely paralyzed and moribund, while most hippocampal neurons appear pyknotic. LSD1 protein is still present in neurons up to 7 weeks post-injection, despite a full deletion of the locus within 24 h [181]. The persistence of LSD1 in induced genetic nulls could be due to a very stable mRNA, a long protein half-life, or both. This finding indicates that the absence of LSD1 protein induces neuronal cell death very rapidly, over the span of just 1 week. Furthermore, mice are sensitive to very small fluctuations in LSD1, since we observe learning and memory deficits as early as 4 weeks post-injection.

Why would an epigenetic factor that orchestrates cell fate transition be required in terminally differentiated neurons? To address this paradox, we examined how loss of LSD1 affects neuronal homeostasis. RNA-seq and immunohistochemistry (IHC) in LSD1-deficient hippocampus revealed an upregulation of several pluripotency transcripts, including *Foxo1, Klf4, Oct4* and *Myc*. This upregulation of pluripotency transcripts is accompanied by a reactivation of cell cycle markers. Through IHC, we determined that these pathways are upregulated specifically in neurons. Thus, it appears that LSD1 contributes to actively maintaining the differentiated state of neurons by preventing the inappropriate expression of genes associated with other cell fates. Remarkably this data hints that neurons are not “locked” into their cell fate. Instead they must continually employ epigenetics mechanisms to actively maintain their differentiated cell fate.

Prior to our study, LSD1 had never before been implicated in neurodegenerative disease. However, we observed a specific correlation between the *Lsd1* ^*CAGG*^ hippocampus transcriptional profiles and those observed in human late-onset Alzheimer’s disease (LOAD) and frontotemporal dementia (FTD) [142, 182]. Specifically, without LSD1 in hippocampal neurons, there is an upregulation of the complement cascade pathway, as well as the microglia and immune transcriptional network. In addition, genes involved in neurotransmission and oxidative phosphorylation are downregulated. These pathways are similarly perturbed in LOAD and FTD patients. Beyond the individual neurodegeneration pathways, the transcriptional changes in *Lsd1* ^*CAGG*^ hippocampus highly correlate with the transcriptional changes in the prefrontal cortex of AD and FTD patients genome wide.

Given the striking neurodegenerative phenotype and transcriptional overlap with human dementias, we hypothesized that LSD1 might be affected in these neurodegenerative diseases. In human LOAD and FTD cases, we find that *LSD1* mislocalizes with pathological Tau aggregates in AD and TDP-43 aggregates in FTD. These findings suggest a new model for these diseases: as the pathological proteins pTau (in AD), or TDP-43 (in FTD), accumulate in aging neurons, *LSD1,* normally localized to the nucleus, becomes sequestered by these protein aggregates in the cytoplasm. Its mislocalization interferes with the neuron’s ability to epigenetically maintain its cell fate by blocking inappropriate transcription, and this inappropriate transcription ultimately leads to the activation of multiple neurodegeneration pathways (Fig. 4b).

Much more work is needed to understand the etiology of LSD1-mediated neurodegeneration. For example, it’s currently unclear if LSD1-deficient neurons die from necrosis, apoptosis, or engulfment in a microglia response pathway. Would inhibiting these pathways prevent cell death in *Lsd1* mutant mice? Understanding the mechanism of neuronal death in these animals is the first step toward blocking death. These data also raise the question of why neurons appear to be the cell type most sensitive to LSD1 loss. The study of LSD1 as an epigenetic regulator in the healthy adult brain will help us understand its pivotal role in this cell type. Further, we present for the first time a novel potential therapeutic pathway for targeting AD progression. Could we harness the LSD1 pathway and suppress tauopathy-related phenotypes by inhibiting LSD1 sequestration to the cytoplasm? Could the consequences of pathology be reversed if LSD1 was redirected to the nucleus or if LSD1 gene targets could be pharmacologically repressed?

Presently, our understanding of epigenetics in neurodegeneration is rapidly expanding. As mentioned previously, the role of REST in the aging brain illuminates another pathway, in addition to LSD1, by which epigenetic modification maintains neuronal health. REST can interact with the CoREST complex, which contains LSD1, so it is possible that LSD1 and REST function together. However, despite the similarities in pathological mechanism, we believe that aberrations in the REST and LSD1 pathways occur independently and operate separately in the aging brain. For example, in AD, REST associates with Aβ in autophagosomes, while LSD1 localizes to NFTs of hyperphosphorylated Tau. Also, the reported REST neurodegeneration phenotype in mice is much less severe than what we observe in our LSD1 mutants, suggesting at a minimum that their functions do not completely overlap.

Many other epigenetic modifications have been implicated in AD and other tauopathies. For example, the APP promoter is hypomethylated in AD, though the overall methylation status of the AD genome is debated [183, 184]. Defects in epigenetic silencing in the presence of pTau have also been reported. Specifically, overexpression of mutant human Tau, associated with familial cases of FTD, results in loss of heterochromatin in *Drosophila* and mouse. Additionally, neurons containing NFTs display significant loss of heterochromatin in human AD and FTD cases [185]. This suggests that epigenetic factors that promote heterochromatin formation could be impaired in the presence of NFTs. Currently, it’s not well understood whether these findings indicate the cause or the effect of the disease state. Untangling the two is particularly difficult, since epigenetic modifications regulate a host of downstream gene networks. However, the manipulation of epigenetic pathways in neurodegenerative models will begin to inform on these possibilities and illuminate new therapeutic targets.

## Conclusions

Though originally thought to be relatively stable, epigenetic modifications are capable of dynamic change, providing the perfect medium by which a post-mitotic neuron can respond to changes. For example, alterations in the DNA methylation across the life of a neuron can bolster plasticity or fine-tune a response to a signaling cascade. As a result, the DNA methylation landscape has quickly emerged as a major player in regulating neuronal gene expression. However, as illustrated above, deviations in DNA methylation can severely hinder neuronal health. Without maintenance from the DNMT proteins, DNA methylation can be completely lost at some gene promoters, altering the transcription of the locus. Alternatively, mutation in MeCP2, the reader of DNA methylation, can change the transcriptional profile of the neuron, severely hindering its plasticity and vitality. In addition, changes in DNA methylation at the FMR1 locus may underlie the etiology of FXS, as the FMR1 transcript acts *in cis* to alter the histone modification profile and increase CpG methylation at its own locus.

As evidenced by the action of both REST and LSD1, histone modifications may also have a profound effect on gene regulation in post-mitotic neurons. Loss of either of these epigenetic factors is detrimental to the health of adult neurons and induces a cascade of transcriptional changes. Further, deletion of LSD1 from post-mitotic neurons causes the cells to undergo rapid neurite degeneration and death. Curiously, before they degenerate, LSD1 deleted neurons reactivate several critical stem cell factors, suggesting that the cells potentially experience a reversion in cell identity. Considering these combined findings, the complexity of neuroepigenetics emerges: some epigenetic modifications must be actively preserved to maintain identity across the long life of the neuron. However, other modifications must be capable of dynamic change to allow for the rapid response of the neuron to its environment. Based on the ongoing function of these neuroepigenetic mechanisms in post-mitotic neurons, it is tempting to speculate that oxidative toxicity, protein aggregates, and/or environmental factors could hinder the ability of genetically “normal” epigenetic players to perform their tasks, compromising a host of downstream pathways necessary for neuronal cell health.

## Final thoughts

Since neurons are post-mitotic, it was not necessarily clear that they would require epigenetic mechanisms that regulate gene transcription. However, we have highlighted a few emerging examples of how epigenetic mechanisms may be functioning in neurological disease. These examples clearly demonstrate the need for epigenetic regulation in the nervous system, but because there are relatively few examples thus far, no unifying mechanisms have yet emerged. Nevertheless, we have discussed cases where the function of epigenetic modifying enzymes may be different in post-mitotic neurons than in dividing cells. In addition, we have highlighted an example of how an epigenetic enzyme that normally functions during cell fate transitions may instead be functioning to maintain the terminally differentiated state of post-mitotic neurons. These examples illustrate the need to think about epigenetic mechanisms in post-mitotic cells differently. Perhaps by doing so, it will enable us to uncover further examples of how altered epigenetic mechanisms may be contributing to neurological disease.

## Abbreviations

DNMT: DNA (cytosine-5) methyltransferase
5mC: 5-methylcytosine
5hmC: 5-hydroxymethylcytosine
5fC: 5-formylcytosine
5caC: 5-carboxylcytosine
TET: ten-eleven translocase
TDG: thymine-DNA glycosylase
INTACT: isolation of nuclei tagged in specific cell types
MLL: mixed lineage leukemia
SET: Su(var)3-9, enhancer of zeste and trithorax
LSD: lysine-specific histone demethylase
Jmj: Jumonji domain
JARID: Jmj/ARID domain-containing demethylase
SUV: suppressor of variegation
EHMT: euchromatic histone–lysine *N*-methyltransferase
EZH2: enhancer of zeste homology 2
PRC: polycomb repressor complex
UTX: ubiquitously transcribed tetratricopeptide repeat X chromosome
Xist: X-inactive specific transcript
YY1: Yin Yang 1
HOTAIR: Hox transcript antisense RNA
piRNA: Piwi-interacting RNA
MECP: methyl-CpG-binding protein
HDAC: histone deacetylase
RTT: Rett syndrome
FXS: Fragile X syndrome
FMR1: Fragile X mental retardation 1
FMRP: Fragile X mental retardation protein
UTR: untranslated region
ESC: embryonic stem cells
ChIRP: chromatin isolation by RNA purification
AD: Alzheimer’s disease
Aβ: beta-amyloid protein
BACE: β-secretase protein
APOE: apolipoprotein E
NFT: neurofibrillary tangles
REST: RE1-silencing transcription factor
CtBP: C-terminal binding protein
FTD: frontotemporal dementia
LBD: Lewy body dementia
NuRD: nucleosome remodeling deacetylase
